# The interaction between the community food environment and cooking skills in association with diet-related outcomes in Dutch adults

**DOI:** 10.1017/S1368980023002148

**Published:** 2023-12

**Authors:** Noreen Z Siddiqui, Maria GM Pinho, Femke Rutters, Joline WJ Beulens, Joreintje D Mackenbach

**Affiliations:** 1 Amsterdam UMC, Location Vrije Universiteit Amsterdam, Epidemiology and Data Science, De Boelelaan 1089a, 1081 HV Amsterdam, the Netherlands; 2 Amsterdam Public Health, Health Behaviors and Chronic Diseases, Amsterdam, the Netherlands; 3 Upstream Team, Amsterdam, the Netherlands; 4 Julius Center for Health Sciences and Primary Care, University Medical Centre, Utrecht, the Netherlands

**Keywords:** Food environment, Cooking skills, Diet-related outcomes, Diet quality, BMI, Frequency of home cooking

## Abstract

**Objective:**

We examined whether associations between the food environment, frequency of home cooking, diet quality and BMI were modified by the level of cooking skills.

**Design:**

Cross-sectional study using linear and modified Poisson regression models adjusted for age, sex, energy intake, education, income, household size and urbanisation. The frequency of home cooking was categorised into <6 and 6–7 d. Diet quality was based on a validated Dutch healthy diet index (0–150 points). Count of restaurants and food stores were determined by their count in a 1000m buffer around home and work. Cooking skills (score 1–5) were assessed using a validated questionnaire and added as interaction term.

**Setting:**

The Netherlands.

**Participants:**

1461 adults aged 18–65 years.

**Results:**

Count of restaurants and food stores were not associated with the frequency of home cooking. A 10-unit higher count of food stores was associated with a higher diet quality (*β*: 0·58 (95 % CI (0·04, 1·12)), and a 10-unit higher count of restaurants was associated with a lower BMI kg/m^2^ (*β*: −0·02 (95 % CI (-0·04, −0·004)). Better cooking skills were associated with a higher likelihood of cooking 6–7 d compared with <6 d (risk ratio: 1·24 (95 % CI (1·16, 1·31)) and a higher diet quality (*β*: 4·45 (95 % CI (3·27, 5·63)) but not with BMI. We observed no interaction between the food environment and cooking skills (*P*-for-interaction > 0·1).

**Conclusions:**

Exposure to food stores was associated with a higher diet quality and exposure to restaurants with a lower BMI. Better cooking skills were associated with a higher frequency of home cooking and better diet quality but did not modify associations with the food environment. Future studies should explore different approaches to understand how individuals interact with their food environment.

A healthy dietary pattern such as one rich in vegetables, fruits, legumes and fibres, and with low consumption of SFA and red meat, is associated with a lower risk of developing obesity^(1)^. Frequency of home cooking and cooking skills are of importance, as previous literature indicated that the frequency of home cooking and better cooking skills were associated with a better diet quality^(2,3)^. The type of food, quantity and the way in which people eat (e.g. healthy or unhealthy foods) result from an interaction of individual-level factors such as taste preferences, budget, cooking skills, promoted, marketed and what is accessible and available in the food environment^(4,5)^.

The food environment is the combination of all social, physical, economic and online aspects that influence food choices and comprises both healthy and unhealthy features, for example (organic) grocery stores or (fast-food) restaurants^(6)^. An often used typology of food environments by Glanz *et al*.^(7)^ is a distinction between the community, consumer and organisational food environment, which are in turn influenced by the information and policy environment. The community food environment, which consists of the types and locations of food outlets in communities, is especially of interest for public health research, because it has changed drastically in the last decades^(8–10)^. This has resulted in drastic increases in the availability and accessibility of food outlets selling ultra-processed, energy-dense foods in many areas^(11)^. Even though the evidence remains contradictive^(12–14)^, easy access to affordable, unhealthy foods is likely to contribute to unhealthier diets and in turn increases the risk of obesity.

Alongside the change in food environments, a transition in cooking and food preparation skills has also been observed^(15)^. This transition has led to an increased use of pre-prepared, packaged and convenience foods, which require fewer and/or different skills than what is often referred to as traditional or ‘from scratch’ cooking^(16)^. Indeed, spending less time per day on food preparation is associated with a more frequent use of take-away and full-service restaurants^(17)^. The decreased time spent on cooking^(18,19)^ is likely attributable to a preference for convenience and competing time-use activities such as socialising.

Previous research has shown that adults with better nutrition knowledge and cooking skills have better quality diets^(20–22)^. We defined cooking skills as the ability to prepare meals in different ways^(23)^. It may be hypothesised that individuals with better nutrition knowledge and cooking skills are better able to find nutritious foods among the ubiquitous availability of unhealthy foods. Alternatively, individuals with better cooking skills may be able to prepare a healthy meal in less time than those without good cooking skills, thereby relying less on ‘convenience’ options in the food environment such as takeaway or fast-food meals.

To date, it remains unclear how the food environment and cooking skills interact in relation to the frequency of home cooking, diet quality and BMI.

Therefore, we aimed to study if a potential association of the food environment with the frequency of home cooking, diet quality and BMI is modified by the levels of cooking skills.

## Methods

### Study design and study population

We used data from the Eet & Leef study, a cross-sectional survey that was designed to explore how food retail environments influence food choices and health. The study design and data collection methods have been outlined previously^(24,25)^. In brief, participants were eligible if they were able to understand the Dutch language, and if they had access to a computer with internet and e-mail address; thus, 2522 eligible participants between the ages of 18 and 65 years from the twenty largest urban cities in the Netherlands were registered and invited to participate in the survey^(25)^. In order to create a diverse study population, the recruitment of participants followed a stepwise approach. Initially, postal invitations were sent at random to home addresses in the twenty largest cities in the Netherlands. In addition, a targeted Facebook and Instagram campaign was implemented to increase the number of men, as well as women with lower education. Finally, invitations were sent to individuals from these subgroups who had previously participated in studies conducted at our department. Participants were asked to complete three parts of a web-based survey on the food environment, dietary intake and health-related variables including BMI. All variables in the current study were derived from the first part of the questionnaire that was completed by all 1784 participants, except for dietary intake that was derived from the third part of the survey that was completed by 1492 participants. We continued our analyses with participants who had complete dietary data available and excluded thirty-one participants because of unlikely energy intake levels: fewer than 500 and greater than 3500 kcal for women and fewer than 800 and greater than 4200 kcal for men^(26)^. This resulted into an analytical sample size of 1461 participants.

Upon completion of the three-part survey, participants received a gift voucher of 15 euros. This study was approved by the Medical Ethical Committee of VU University Medical Center (no. 2019.307); all participants provided digital informed consent.

### Exposures

#### Food environment

Data regarding the geographical location and types of food retailers from 2019 were gathered from Locatus^(27)^, a commercial company from the Netherlands. This Dutch commercial dataset has previously been validated and showed ‘excellent’ agreement for both the location and classification of food outlets with a *κ* of 0·953 and a concordance of 0·939^(28)^. Data from Locatus were linked to the specific home addresses and to the work addresses. We defined the main exposure variables as the count of food stores per 10-unit higher count around the home and work environment that sell ingredients to cook, such as a supermarkets, greengrocers, bakeries, poultries and butchers. We included these types of food retailers, because they mostly sell ingredients to be prepared at home^(29)^. In addition, we calculated the count of restaurants per 10-unit higher count around the home and work environment that sell prepared meals, such as restaurants and fast-food shops, since these places mostly prepare meals to eat away from home^(29)^. We analysed our results per 10-unit higher count to facilitate the interpretations of the results.

#### Cooking skills

We had data of six items about cooking skills on a 5-point Likert scale from the Food Literacy questionnaire developed by Poelman *et al.* ^(30)^ to assess cooking skills. Confirmatory factor analysis could not confirm the proposed factor structure of the six items loading onto one factor (Comparative Fit Index = 0·97, Root Mean Square Error of Approximation = 0·07, Tucker-Lewis Index = 0·95). Exploratory factor analysis suggested that one item needed to be removed (‘Are you able to change a meal? For example if you are missing one of the ingredients?’) and that the remaining five items loaded onto one factor. These were questions regarding (1) the preparation of fresh vegetables in different methods; (2) if the participant finds it hard to prepare a meal with more than five ingredients; (3) if the participant is able to make alterations to the meal (in case there is an ingredient missing); (4) whether the participant is able to prepare fresh fish in different ways and (5) and if the participant can prepare a meal with fresh ingredients ‘from scratch’. Internal consistency of those items was good (Cronbach’s *α* in this study: 0·77), and test–retest reliability was good (Spearman’s correlation of 0·84). The level of cooking skills used in our analytical sample consisted out of an average score that was calculated and ranged from 1 to 5, where a higher score indicated a better level of cooking skills.

### Outcomes

#### Frequency of home cooking

In order to evaluate the frequency of home cooking, the participants were asked to fill in how often they or their partner cooks at home. The following answering options were available: ‘never’; ‘1–2 times per year’; ‘5–6 times per year’; ‘1 time per month’; ‘2 times per month’; ‘1 time per week’; ‘2 times per week’; ‘3 times per week’; ‘4 times per week’; ‘5 times per week’; ‘6 times per week’ or ‘every day’. For our analyses, we dichotomised the frequency of home cooking into cooking <6 and 6–7 d a week, to create a more equal data distribution between groups.

#### Diet quality

Dietary intake was estimated from a 34-item FFQ – the Dutch Healthy Diet FFQ. This FFQ was used to assess adherence to the Dutch Dietary guidelines from 2015. Based on the FFQ, fifteen components of the Dutch Healthy Diet Index of 2015 (DHD15-index) were derived and were used to study diet quality in our study population. This index ranged from 0 to 150 with the highest index indicating a better diet quality according to the Dutch dietary guidelines^(31)^. Validation of the DHD15-index derived from the Dutch Healthy Diet FFQ was considered acceptably correlated with the DHD15-index derived from the reference method, the 180-itemed FFQ, with a Spearman’s correlation of 0·57 (95 % CI (0·53·0·60))^(31)^.

#### BMI

Participants were asked to fill in their length in centimetres and their weight in kilograms. We calculated BMI by dividing weight by the square of height (weight (kg)/height (m^2^)).

#### Covariates

Information regarding sex and age was self-reported. Educational level of the participant was asked in eight different categories according to the Dutch educational system: no education; primary school; lower vocal education; general secondary education; secondary vocational education; higher general secondary education; higher professional education and not applicable/do not know. Net income of the participants’ household per month was asked and six different answering options were available, with a range between 0 and 1200 euros per month and more than 4000 euros per month. Household composition was defined by the number of adults and children living in one house. Information regarding kilocalories (kcal) was derived from the FFQ. Information about urbanisation was retrieved from the Central Bureau of Statistics Netherlands and coded as (1) very urban (>2500 addresses per km^2^); (2) strongly urban (1500–2500 addresses per km^2^); (3) moderately urban (1000–1500 addresses per km^2^); (4) little urban (500–1000 addresses per km^2^) and (5) not urban (<500 addresses per km^2^)^(32)^.

### Statistical analyses

Multivariable linear and modified Poisson regression analyses were performed to analyse associations between the count of restaurants and food stores and diet-related outcomes. We decided to use modified Poisson regression models instead of logistic regression models, because odds ratios overestimate risk ratios when the event (home cooking 6–7 times a week) is common^(33)^. Modified Poisson regression analyses produce risk ratios; however, the frequency of home cooking is not a public health risk. Therefore, instead of interpreting our results in risk ratios, we interpreted our results in terms of likelihood to facilitate the interpretations.

We tested linearity assumptions using quadratic terms, but the model fit did not improve between the independent variable and any of the outcome variables when including these terms.

We developed three models to study associations between our primary exposures, count of restaurants and count of food stores per 10-unit higher count and our three different outcomes: frequency of home cooking, diet quality and BMI. For our first outcome, frequency of home cooking, we performed modified Poisson regression analyses where we adjusted for sex, age and energy intake (kcal) in our first model. In our second model, we additionally adjusted for educational level, net household income, household composition and urbanisation. In our third model, we added level of cooking skills as an interaction term to the fully adjusted model (model 2). For our second and third outcomes, diet quality and BMI, we performed linear regression analyses and used the same models. Analyses were stratified when an interaction (*P*-for-interaction <0·1) was obtained for cooking skills.

In addition, we also studied associations between cooking skills and frequency of home cooking, diet quality and BMI, where we adjusted for the same confounders in model 1 and 2 as described earlier.

We excluded individuals with missing exposure data and dietary information and used multiple imputation (m = 10 imputations) to impute missing values of all other covariates except for energy intake (0·3 % education – 7·7 % net household income), based on the fully conditional specification methods (predictive mean matching) to prevent reduced power and attrition bias^(34)^. Pooled estimated results based on imputed data were used in the regression models. We used R statistics version 4.0.3 for all statistical analyses.

## Results

We included 1461 participants in our study population with a mean age of 42·5 (±13·7) years. Around 64·1 % of the participants were women and 57·1 % had a higher education. Further descriptive statistics are presented in Table 1.

**Table 1.** Descriptive characteristics of included participants from the Eet & Leef Study participants, N = 1,461

Age (years)	42.5 (+/- 13.7)
Sex (% women)	64.1
Educational level (% higher professional education)	57.1
Net household income (% more than 4000 euros per month)	25.3
Count of restaurants around the home and work environment	33.0 (12.0, 122.0)
Count of food stores around the home and work environment	14.0 (6.0, 26.0)
Level of cooking skills (score 1-5)	4.0 (3.4, 4.6)
DHD15-index (range 0-150) – diet quality indicator	96.3 (**+/**- 18.4)
Frequency of home cooking (% cooking less than 6 times a week)	36.8
BMI (kg/m^2^)	24.8 (**+/**- 4.6)
Energy intake (kcal)	1,521 (**+/**- 512.7)
Household composition (members of a household)	1.5 (1.3-2.1)
Urbanization (% individuals living in high urbanized areas (buffer areas with >2,500 addresses per km^2^))	45.2

Descriptive statistics of characteristics of the study participants from the Eet & Leef Study based om imputed data of covariates (m = 10). Variables are presented in percentages (%), variables with a normal distribution are presented as standard deviations (+/-), variables with a skewed distribution are presented as median with an interquartile range (IQR).

Table 2 shows the risk ratios per 10-unit higher count of restaurant and food stores around the home and work environment and the frequency of home cooking. No associations were found between the count of restaurants and frequency of home cooking (risk ratio: 1·00 (95 % CI (0·99, 1·00)). Similarly, no associations were found between count of food stores and frequency of home cooking (risk ratio: 0·99 (95 % CI (0·97, 1·02)). We found no interaction between count of restaurants or food stores and cooking skills in relation to frequency of home cooking (*P*-for-interaction: 0·7 and 0·9, respectively).

**Table 2:** Main results of restaurants/food stores around the home and work environment and diet-related outcomes

		Frequency of home cooking (cooking <6 d a week) (*n* 1461)		Diet quality index (0–150) (*n* 1461)		BMI (kg/m^2^) (*n* 1461)	
		RR	95 % CI	RR	95 % CI	RR	95 % CI
Per 10-unit count of restaurants around the home and work environment	Model 1*	1·00	0·99, 1·00	**0·09**	**0·02, 0·16**	**−0·03**	**-0·05, −0·01**
	Model 2†	1·00	0·99, 1·00	0·05	−0·02, 0·11	**−0·02**	**-0·04, −0·004**
Per 10-unit count of food stores around the home and work environment	Model 1	0·98	0·96, 1·01	**0·75**	**0·26, 1·24**	−0·08	−0·21, 0·04
	Model 2	0·99	0·97, 1·02	**0·58**	**0·04, 1·12**	−0·03	−0·17, 0·11

* Model 1: adjusted for sex, age and energy intake (kcal).

† Model 2: adjusted for model 1, and educational level, net household income, household composition and urbanisation. Statistical significance (*P* < 0·05) is indicated in bold font.

Results are presented in risk ratios (RR) and 95 % confidence intervals obtained from modified Poisson regression analyses to study associations between restaurants and food stores around the home and work environment and frequency of home cooking (cooking <6 d a week). Results are presented in beta coefficients (*β*) and 95 % confidence intervals obtained from linear regression analyses to study associations between restaurants and food stores around the home and work environment with outcomes diet quality index (0–150) and BMI (kg/m^2^) in the general population. Based on imputed data of covariates (m = 10).

We also observed no association in the second model between a 10-unit higher count of restaurants and diet quality (*β* 0·05 (95 % CI (-0·02, 0·11) see Table 2). However, a 10-unit higher count of food stores was associated with a higher diet quality in model 2 (*β* 0·58 (95 % CI (0·04, 1·12)). We found no interaction between count of restaurants or food stores and cooking skills in relation to diet quality (*P*-for-interaction: 0·1 and 0·5, respectively).

In addition, Table 2 shows that, per 10-unit higher count of restaurants, a lower BMI was found (*β* −0·02 (95 % CI (-0·04, −0·004)). No associations were found for count of food stores in model 2 (*β* −0·03 (95 % CI (-0·17, 0·11)). Again, we found no interaction between count of restaurants or food stores and cooking skills in relation to BMI (*P*-for-interaction: 0·8 and 0·9, respectively).

In additional analyses, we observed that better cooking skills were associated with a higher likelihood of cooking 6–7 times a week, compared with <6 d a week (model 1: risk ratio: 1·25 (95 % CI (1·18, 1·33)), model 2: risk ratio: 1·24 (95 % CI (1·16, 1·31))) and a higher diet quality (model 1: *β* 5·60 (95 % CI (4·42, 6·80)), model 2: *β* 4·45 (95 % CI (3·27, 5·63))) but not with BMI, see Supplemental Table 1.

## Discussion

We examined whether the association between the food environment around home and work and the frequency of home cooking, diet quality and BMI was modified by the level of cooking skills among Dutch adults. We expected that individuals with better cooking skills would rely less on the food environment and would therefore have better diet-related outcomes even when exposed to an unhealthy food environment. However, we found no evidence for effect modification in the association between exposure to food retailers and diet-related outcomes by the level of cooking skills. Associations between the presence of restaurants and food stores around the home and work environment and frequency of home cooking, diet quality and BMI were mixed, since we found a positive association between better cooking skills and a higher likelihood of frequency of home cooking and a higher diet quality, but not with BMI.

To our knowledge, no studies have investigated effect modification by the level of cooking skills in association with the food environment. Previous studies did however show other types of individual effect modifiers, such as socioeconomic status (SES)^(35)^, self-control^(36)^ and financial strain^(37)^, but also found limited evidence for effect modification. This may be explained by usage of different measures for the food environment (e.g. using proximity or density measures) or because effect modification by personal characteristics does not have a substantial influence.

It could be speculated that the lack of moderating effects in this and other studies is due to the inconsistent main effects of food environment exposures and diet-related outcomes^(12,13,38,39)^. Indeed, while we did find that a higher availability of food stores selling ingredients for meals around home and work was associated with a better diet quality, we also observed that a higher availability of restaurants around home and work was associated with a lower BMI, although effect sizes were very small. More importantly, we found no meaningful associations between either availability of food stores or availability of restaurants with frequency of home cooking.

There are several explanations for these null or inconsistent results that have also been outlined elsewhere^(25,38,40)^, such as self-selection bias^(13)^, reverse causality^(41)^, the co-location of healthy and unhealthy food retailers^(42)^, interaction with other built environment factors such as walkability^(43)^, interaction with other food environment factors such as affordability^(44)^ and lack of mediating variables such as actual use of the food retailers under investigation^(45)^. In addition, our participants had a relatively high level of cooking skills and high frequency of home cooking, which may also have masked any results that may be observed in a population less skilled in cooking.

We observed that better cooking skills were associated with a higher frequency of home cooking and better diet quality, which is in line with previous studies^(22,46,47)^. We did not find associations between cooking skills and BMI, but this may be due to self-selection bias where young individuals with lower BMI live in urban areas with many restaurants^(48)^. It may also be attributable to factors related to the other side of the energy balance (i.e. physical activity). Not many studies have investigated this association between cooking skills and BMI before; therefore, we cannot confirm whether there is evidence that cooking skills and BMI are associated with each other. All in all, the question remains whether better cooking skills can ‘protect’ individuals from an unhealthy food environment, and we hypothesise that this is because of the difficulties in defining ‘true exposure’ to the food environment. It would be of interest to use methodologies to measure food environment exposure more precisely, such as Global Positioning Systems^(49)^, and combine this with survey data on which food outlets were actually used.

The results of this study should be interpreted in the light of some strengths and limitations. A main strength of this study is the extensive database with a range of variables on diet-related outcomes and psychosocial resources, such as cooking skills that could be linked to individuals’ home and work addresses. The latter is especially a strong point of the study, since most studies only use exposure to food outlets around the home, which underestimates the ‘true exposure’^(11)^. Yet, future studies may want to include additional covariates such as cultural background and type of employment, which could influence the time that individuals spend on home cooking as described earlier^(2)^.

An important limitation of this study is the cross-sectional nature: changing food environments are very likely to have a more important role for the increased prevalence of obesity than that current food environments can explain individual variations in BMI. However, to the best of our knowledge, no studies have data on changes in food environment exposure, changes in diet-related outcomes and cooking skills available.

In addition, we used self-reported BMI data, which could have led to underestimations of participants’ BMI, although previous studies have demonstrated a relatively good validity of BMI self-reports^(50)^. Another limitation that should be discussed is the use of a shorter version of the FFQ with 34-items compared with the full-length FFQ of 180-items^(31)^. Although van Lee *et al*.^(31)^ validated the 34-itemed FFQ against the 180-itemed FFQ and reported that the DHD-FFQ had acceptable ranking capacity in individuals, this may partially explain the lower-than-average mean energy intake in our sample. Although we found some significant associations, the effect sizes were small. Therefore, translations to practice may be limited. Especially, since effect sizes were presented as an increase per 10-unit higher count in food stores or restaurants within a 1000-m buffer. Finally, we speculate that the lack of effect modification may be attributable to the lack of consistent main effects. Therefore, it would be interesting for future studies to explore different methodologies to study the interaction between individuals and their food environment.

## Conclusions

We conclude that being exposed to food stores was associated with a higher diet quality and being exposed to restaurants was associated with a lower BMI. Better cooking skills were associated with a higher frequency of home cooking and a better diet quality but did not modify the observed associations with the food environment. Future studies should explore different approaches to understand how individuals interact with their food environment.

## Supporting information

Siddiqui et al. supplementary material Siddiqui et al. supplementary material

## Supplementary material

For supplementary material accompanying this paper visit https://doi.org/10.1017/S1368980023002148

## References

[1] Seiler A , Chen MA , Brown RL et al. (2018) Obesity, dietary factors, nutrition, and breast cancer risk. Curr Breast Cancer Rep 10, 14–27. 30662586 10.1007/s12609-018-0264-0 PMC6335046

[2] Mills S , White M , Brown H et al. (2017) Health and social determinants and outcomes of home cooking: a systematic review of observational studies. Appetite 111, 116–134. 28024883 10.1016/j.appet.2016.12.022

[3] Tani Y , Fujiwara T & Kondo K (2020) Cooking skills related to potential benefits for dietary behaviors and weight status among older Japanese men and women: a cross-sectional study from the JAGES. Int J Behav Nutr Phys Act 17, 82. 32590984 10.1186/s12966-020-00986-9 PMC7318755

[4] Kremers SP , de Bruijn GJ , Visscher TL et al. (2006) Environmental influences on energy balance-related behaviors: a dual-process view. Int J Behav Nutr Phys Act 3, 9. 16700907 10.1186/1479-5868-3-9 PMC1481572

[5] Mackenbach JD , Lakerveld J , Van Lenthe FJ et al. (2016) Interactions of individual perceived barriers and neighbourhood destinations with obesity-related behaviours in Europe. Obes Rev 17, Suppl. 1, 68–80. 10.1111/obr.12374 26879115

[6] Burgoine T & Monsivais P (2013) Characterising food environment exposure at home, at work, and along commuting journeys using data on adults in the UK. Int J Behav Nutr Phys Act 10, 85. 23806008 10.1186/1479-5868-10-85 PMC3720205

[7] Glanz K , Sallis JF , Saelens BE et al. (2005) Healthy nutrition environments: concepts and measures. Am J Health Promot 19, 330–333. 15895534 10.4278/0890-1171-19.5.330

[8] James P , Seward MW , James O’Malley A et al. (2017) Changes in the food environment over time: examining 40 years of data in the Framingham Heart Study. Int J Behav Nutr Phys Act 14, 84. 28646894 10.1186/s12966-017-0537-4 PMC5483254

[9] Pinho MGM , Mackenbach JD , den Braver NR et al. (2020) Recent changes in the Dutch foodscape: socioeconomic and urban-rural differences. Int J Behav Nutr Phys Act 17, 43. 32197651 10.1186/s12966-020-00944-5 PMC7083034

[10] Hobbs M , Mackenbach JD , Wiki J et al. (2021) Investigating change in the food environment over 10 years in urban New Zealand: a longitudinal and nationwide geospatial study. Soc Sci Med 269, 113522. 33339682 10.1016/j.socscimed.2020.113522

[11] Monteiro CA , Moubarac JC , Cannon G et al. (2013) Ultra-processed products are becoming dominant in the global food system. Obes Rev 14, Suppl. 2, 21–28. 24102801 10.1111/obr.12107

[12] Caspi CE , Sorensen G , Subramanian SV et al. (2012) The local food environment and diet: a systematic review. Health Place 18, 1172–1187. 22717379 10.1016/j.healthplace.2012.05.006 PMC3684395

[13] Cobb LK , Appel LJ , Franco M et al. (2015) The relationship of the local food environment with obesity: a systematic review of methods, study quality, and results. Obesity 23, 1331–1344. 26096983 10.1002/oby.21118 PMC4482774

[14] Holsten JE (2009) Obesity and the community food environment: a systematic review. Public Health Nutr 12, 397–405. 18477414 10.1017/S1368980008002267

[15] Lam MCL & Adams J (2017) Association between home food preparation skills and behaviour, and consumption of ultra-processed foods: cross-sectional analysis of the UK national diet and nutrition survey (2008–2009). Int J Behav Nutr Phys Act 14, 68. 28535769 10.1186/s12966-017-0524-9 PMC5442685

[16] James WP (2008) The epidemiology of obesity: the size of the problem. J Intern Med 263, 336–352. 18312311 10.1111/j.1365-2796.2008.01922.x

[17] Monsivais P , Aggarwal A & Drewnowski A (2014) Time spent on home food preparation and indicators of healthy eating. Am J Prev Med 47, 796–802. 25245799 10.1016/j.amepre.2014.07.033 PMC4254327

[18] Smith LP , Ng SW & Popkin BM (2013) Trends in US home food preparation and consumption: analysis of national nutrition surveys and time use studies from 1965–1966 to 2007–2008. Nutr J 12, 45. 23577692 10.1186/1475-2891-12-45 PMC3639863

[19] Plessz M & Etile F (2019) Is cooking still a part of our eating practices? Analysing the decline of a practice with time-use surveys. Cult Sociol 13, 93–118.

[20] Hasan B , Thompson WG , Almasri J et al. (2019) The effect of culinary interventions (cooking classes) on dietary intake and behavioral change: a systematic review and evidence map. BMC Nutr 5, 29. 32153942 10.1186/s40795-019-0293-8 PMC7050805

[21] Carbonneau E , Lamarche B , Provencher V et al. (2021) Associations between nutrition knowledge and overall diet quality: the moderating role of sociodemographic characteristics-results from the PREDISE study. Am J Health Promot 35, 38–47. 32515200 10.1177/0890117120928877

[22] Lavelle F , Bucher T , Dean M et al. (2020) Diet quality is more strongly related to food skills rather than cooking skills confidence: results from a national cross-sectional survey. Nutr Diet 77, 112–120. 31602753 10.1111/1747-0080.12583

[23] Hartmann C , Dohle S & Siegrist M (2013) Importance of cooking skills for balanced food choices. Appetite 65, 125–131. 23402717 10.1016/j.appet.2013.01.016

[24] Hoenink JC , Waterlander W , Beulens JWJ et al. (2022) The role of material and psychosocial resources in explaining socioeconomic inequalities in diet: a structural equation modelling approach. SSM Popul Health 17, 101025. 35097184 10.1016/j.ssmph.2022.101025 PMC8783096

[25] Mackenbach JD , Hobbs M & Pinho MG (2022) Where do Dutch adults obtain their snack foods? Cross-sectional exploration of individuals’ interactions with the food environment. Health Place 75, 102802. 35462182 10.1016/j.healthplace.2022.102802

[26] Banna JC , McCrory MA , Fialkowski MK et al. (2017) Examining plausibility of self-reported energy intake data: considerations for method selection. Front Nutr 4, 45. 28993807 10.3389/fnut.2017.00045 PMC5622407

[27] Locatus Retail Data. https://locatus.com/en/retail-data/ (accessed August 2021).

[28] Canalia C , Pinho MGM , Lakerveld J et al. (2020) Field validation of commercially available food retailer data in the Netherlands. Int J Environ Res Public Health 17, 1946. 32188152 10.3390/ijerph17061946 PMC7143735

[29] Lake AA , Burgoine T , Greenhalgh F et al. (2010) The foodscape: classification and field validation of secondary data sources. Health Place 16, 666–673. 20207577 10.1016/j.healthplace.2010.02.004

[30] Poelman MP , Dijkstra SC , Sponselee H et al. (2018) Towards the measurement of food literacy with respect to healthy eating: the development and validation of the self perceived food literacy scale among an adult sample in the Netherlands. Int J Behav Nutr Phys Act 15, 54. 29914503 10.1186/s12966-018-0687-z PMC6006995

[31] van Lee L , Feskens EJ , Meijboom S et al. (2016) Evaluation of a screener to assess diet quality in the Netherlands. Br J Nutr 115, 517–526. 26628073 10.1017/S0007114515004705

[32] Bresters P (2019) Toelichting Wijk- en Buurtkaart (Explanation of the Neighborhood Map) 2017, 2018 en 2019. The Hague: Statistics Netherlands.

[33] Zou G (2004) A modified Poisson regression approach to prospective studies with binary data. Am J Epidemiol 159, 702–706. 15033648 10.1093/aje/kwh090

[34] Lewin A , Brondeel R , Benmarhnia T et al. (2018) Attrition bias related to missing outcome data: a longitudinal simulation study. Epidemiology 29, 87–95. 28926372 10.1097/EDE.0000000000000755

[35] Mackenbach JD , Nelissen KGM , Dijkstra SC et al. (2019) A systematic review on socioeconomic differences in the association between the food environment and dietary behaviors. Nutrients 11, 2215. 31540267 10.3390/nu11092215 PMC6769523

[36] Mackenbach JD , Lakerveld J , Generaal E et al. (2019) Local fast-food environment, diet and blood pressure: the moderating role of mastery. Eur J Nutr 58, 3129–3134. 30426195 10.1007/s00394-018-1857-0 PMC6842338

[37] Mackenbach JD , Beenackers MA , Noordzij JM et al. (2019) The moderating role of self-control and financial strain in the relation between exposure to the food environment and obesity: the GLOBE study. Int J Environ Res Public Health 16, 674. 30823592 10.3390/ijerph16040674 PMC6406643

[38] Bivoltsis A , Cervigni E , Trapp G et al. (2018) Food environments and dietary intakes among adults: does the type of spatial exposure measurement matter? A systematic review. Int J Health Geogr 17, 19. 29885662 10.1186/s12942-018-0139-7 PMC5994245

[39] Lytle LA & Sokol RL (2017) Measures of the food environment: a systematic review of the field, 2007–2015. Health Place 44, 18–34. 28135633 10.1016/j.healthplace.2016.12.007

[40] Lytle LA (2009) Measuring the food environment: state of the science. Am J Prev Med 36, S134–S144. 19285204 10.1016/j.amepre.2009.01.018 PMC2716804

[41] Paulitsch RG & Dumith SC (2021) Is food environment associated with body mass index, overweight and obesity? A study with adults and elderly subjects from southern Brazil. Prev Med Rep 21, 101313. 33604235 10.1016/j.pmedr.2021.101313 PMC7876564

[42] Morland K , Wing S , Diez RA et al. (2002) Neighborhood characteristics associated with the location of food stores and food service places. Am J Prev Med 22, 23–29. 11777675 10.1016/s0749-3797(01)00403-2

[43] Tseng M , Thornton LE , Lamb KE et al. (2014) Is neighbourhood obesogenicity associated with body mass index in women? Application of an obesogenicity index in socioeconomically disadvantaged neighbourhoods. Health Place 30, 20–27. 25155451 10.1016/j.healthplace.2014.07.012

[44] Mackenbach JD , Burgoine T , Lakerveld J et al. (2017) Accessibility and affordability of supermarkets: associations with the DASH diet. Am J Prev Med 53, 55–62. 28336352 10.1016/j.amepre.2017.01.044 PMC5478361

[45] Mackenbach JD , Charreire H , Glonti K et al. (2019) Exploring the relation of spatial access to fast food outlets with body weight: a mediation analysis. Environ Behav 51, 401–430.

[46] Farmer N , Wallen GR , Yang L et al. (2019) Household cooking frequency of dinner among non-Hispanic black adults is associated with income and employment, perceived diet quality and varied objective diet quality, HEI (healthy eating index): NHANES analysis 2007–2010. Nutrients 11, 2057. 31480746 10.3390/nu11092057 PMC6769568

[47] Wolfson JA , Leung CW & Richardson CR (2020) More frequent cooking at home is associated with higher Healthy Eating Index-2015 score. Public Health Nutr 23, 2384–2394. 31918785 10.1017/S1368980019003549 PMC11374573

[48] Statistics Netherlands (2018) 100 thousands adults with morbid obesity. https://www.cbs.nl/nl-nl/nieuws/2018/27/100-duizend-volwassenen-hebben-morbide-obesitas#:∼:text=Bij%20een%20BMI%20van%2030,wordt%20gesproken%20van%20morbide%20obesitas (accessed August 2022).

[49] Poelman MP , van Lenthe FJ , Scheider S et al. (2020) A smartphone app combining global positioning system data and ecological momentary assessment to track individual food environment exposure, food purchases, and food consumption: protocol for the observational foodtrack study. JMIR Res Protoc 9, e15283. 32012100 10.2196/15283 PMC7013628

[50] Hodge JM , Shah R , McCullough ML et al. (2020) Validation of self-reported height and weight in a large, nationwide cohort of U.S. adults. PLOS ONE 15, e0231229. 32282816 10.1371/journal.pone.0231229 PMC7153869

