# Supplementary material for: The interaction between the community food environment and cooking skills in association with diet-related outcomes in Dutch adults
Source: Public Health Nutr. 2023 Oct 18;26(12):2945–52. doi: 10.1017/S1368980023002148 (PMC10755447; doi:10.1017/S1368980023002148)
Supplement: Siddiqui et al. supplementary material [file S1368980023002148sup001.docx]

**Supplemental Table 1.** Additional results examining associations between cooking skills and diet-related outcomes.

|  | Level of cooking skills (1-5) | |
| --- | --- | --- |
|  | Model 1 | Model 2 |
| Frequency of home cooking (6-7 days a week) | **RR: 1.25 (95% CI: 1.18, 1.33)** | **RR: 1.24 (95% CI: 1.16, 1.31)** |
| Diet quality index (0-150)^1^ | **β 5.60 (95% CI: 4.42, 6.80)** | **β 4.45 (95% CI: 3.27, 5.63)** |
| BMI (kg/m^2^) | β -0.18 (95% CI: -0.49, 0.14) | β 0.03 (95% CI: -0.29, 0.34) |
| Results are presented in risk ratios (RR), beta coefficients (β) and 95% confidence intervals obtained from modified Poisson and linear regression analyses to study associations between cooking skills in the general population with outcome frequency of home cooking, diet quality, and BMI (kg/m^2^), N = 1,461. Based on imputed data of covariates (m = 10).  Model 1: adjusted for sex, age, energy intake (kcal).  Model 2: adjusted for model 1, and educational level, net household income, household composition, and urbanization. | | |
